# Supplementary material for: Are carbon emissions trading and green financial instruments synergistic? -Comprehensive quantitative research based on content analysis
Source: PLoS One. 2024 Mar 7;19(3):e0298601. doi: 10.1371/journal.pone.0298601 (PMC10919601; doi:10.1371/journal.pone.0298601)
Supplement: S1 Data — (ZIP) [file pone.0298601.s001.zip › Data/地级市Z_F工作报告.zip!/╔╜╬≈/╘╦│╟╩╨/└┤╫╘╣1⁄2╓┌║┼í╛┬φ┐╦╩2╛▌═°í┐_2018.docx]

各位代表：

现在，我代表市人民政府向大会报告工作，请予审议，并请市政协委员和列席人员提出意见。

一、2017年工作回顾

2017年是运城发展史上极不平常的一年，是全市经济发展实现由“疲”转“兴”重大转折的一年，是全市各项工作谋新篇、开新局的一年。一年来，我们认真贯彻党的十八大、十九大精神，坚持以习近平新时代中国特色社会主义思想为指导，在市委的坚强领导下，全面落实省委“一个指引、两手硬”工作思路和要求，按照“改革抢先机，发展站前列，各项工作创一流”总要求，以“走进新时代，建设大运城”为总抓手，统筹推进稳增长、促改革、调结构、惠民生、防风险各项工作，全市经济社会发展取得了新成效。全年地区生产总值完成1336.1亿元，增长7%；规模以上工业增加值完成302.7亿元，增长8.9%；固定资产投资完成616.1亿元，增长6.1%；财政总收入完成129.6亿元，增长22.2%；一般公共预算收入完成67.1亿元，增长13.5%；社会消费品零售总额完成752.8亿元，增长6.8%；外贸进出口总额完成90.8亿元，增长12.5%，其中出口额完成25.6亿元，增长19.4%；城镇居民人均可支配收入完成27302元，增长6.5%；农村居民人均可支配收入完成9992元，增长6.7%，基本实现了全市经济增长保持中高速、全省排位保持中上游的目标。

一是聚力实体经济，工业经济转型步伐全面提速。组织实施“龙腾虎跃”“群星灿烂”“凤还巢”三个发展计划，推动国家级铝镁合金产业、中西部精品钢、汽车及新能源汽车“三大基地”建设。传统产业改造升级力度加大。实施119个技改项目，完成投资68亿元。新兴产业快速发展。全市战略性新兴产业工业增加值增长7.1%，占规上工业比重达到11.7%。新材料、新能源汽车、节能环保、新一代信息技术保持两位数增长。骨干企业成长迅速。86户“龙虎榜”培育企业完成工业总产值1305亿元，占规上工业总产值的77.9%。大运汽车、山西建龙年销售收入首次超过百亿元，实现了运城百亿企业零的突破。新建1个国家级、3个省级中小微企业“双创”基地，净增“小升规”企业26户，创办孵化中小微企业3491户。创新能力不断增强。大禹生物、寰烁科技、寰烁教育3家院士专家工作站挂牌成立，院士专家工作站达到7家，博士后科研工作站达到6家。国家级企业技术中心达到4家，省级39家。高新技术企业新增18家，达到95家。新申请发明专利489件，有效发明专利拥有量754件，中国驰名商标达到28件，地理标志商标达到11件，保持全省领先。全省首家建成并投运的科技大市场年技术合同交易额9.4亿元。大运汽车、亚宝药业通过工信部“两化”融合管理体系认定，大运汽车入选工信部智能制造试点示范，企业数字化、智能化水平进一步提升。

二是聚力提质增效，“三农”工作成效显著。农业生产再获丰收。全年粮食总产30.9亿公斤，小麦亩产创历史新高。蔬菜产量27亿公斤，增长4.5%。水果产量61.7亿公斤，增长6.2%。畜禽肉类总产1.9亿公斤，增长5.7%。主要农作物耕种收综合机械化率达到72.5%。省级优质农产品出口平台建设取得新突破。农产品标准化生产、检验检疫、出口贸易营销“三大体系”建设加速推进。成功举办第二届山西（运城）国际果品交易博览会，推出“运城苹果”区域公用品牌。水果出口3.7亿公斤，增长130%，出口国家和地区达到54个。农民增收渠道不断拓宽。全市外出务工人员达到58.8万人，每年工资性收入200亿元，已成为农村家庭收入的重要来源。脱贫攻坚成效显著。完成228个贫困村退出、7.8万贫困人口脱贫，闻喜、夏县贫困县退出工作完成省级第三方评估验收。农村人居环境有效改善。以“四治六化一创”为重点，集中整治“脏、乱、差、污、绿”问题，农村环境面貌焕然一新。

三是聚力文旅融合，服务业发展势头强劲。大力发展文化旅游、现代金融、商贸物流和生产性服务业，全面提升服务业发展水平。文化旅游业快速提升。成功举办“花之海·俏运城”美丽乡村游、关公国际文化旅游节等重大活动。积极参加“人说山西好风光”第二季城市竞演活动。完成13家旅游景区体制机制改革，实现企业化运营。水峪口、印象风陵、袁家村·运城印象等重点旅游项目推进迅速。全年接待游客人次和实现旅游总收入分别增长26%和28.1%。文化产业增加值占GDP的比重位居全省前列。金融业稳步推进。金融业增加值占GDP的比重达到6.1%。设立了50亿元山西钢铁产业结构调整基金、25亿元大运新能源汽车产业基金、4亿元运芮医药创业投资基金，积极支持产业转型发展。银行信贷平稳增长，存贷比实现了近年来首次回升。“新三板”挂牌企业新增4家，累计达到13家，位居全省第2。着力化解企业担保链难题，垣曲五龙、永济忠民、新绛明达等多家企业债务风险基本化解。信息服务业发展卓有成效。智慧云平台及智慧政务、智慧教育、智慧食药、智慧医疗、智慧城管建成投用。大数据产业园开建，数字运城迈出坚实步伐。

四是聚力规划引领，大运城建设铺开新局。坚持“规划至上、生态宜居、精致建设、特色鲜明、片区开发、管理上档”理念，积极构建“一主三副七个大县城71个建制镇”的城镇化格局。科学确定“一区五带”产业发展总布局，启动编制城镇化、产业发展、重大基础设施、生态环境治理、重大资源开发利用、全域旅游发展“六个专项规划”和新一轮《城市总体规划》。全面推进盐临夏一体化和临猗、夏县撤县设区工作。实施了道路、绿化、城中村和棚户区改造等一批城建工程和总投资108亿元的44个城镇化项目。全市新增集中供热面积740万平方米，中心城区集中供热二期、市民服务中心、文化艺术中心等重点工程有序推进。“六城联创”扎实开展，我市入选住建部第二批全国城市设计试点城市，被省政府授予全省唯一“食品安全示范城市”称号。

五是聚力改革开放，发展活力有效释放。重点改革实现突破。抢抓改革先机，全面深化39项改革任务，十大领域重点改革取得重大进展，一批改革事项走在全省前列。开发区改革顺利推进，5个省级开发区和25个产业园区整合为15个开发区，运城经济技术开发区、盐湖工业园区扩区和闻喜、河津、永济开发区设立获批，“三化三制”稳步推进，工业企业不断向园区集聚。国企国资改革不断深化。运城经济技术开发区投资项目承诺制试点顺利运行，市政府下放职权479项。实施“多证合一、一照一码”，推行企业名称核准全程电子化、网上登记注册等制度，市场主体总量和增速保持全省领先。供销社综合改革成效显著，闻喜惠农服务中心模式在全省推广。城乡义务教育一体化改革进展顺利，全面通过全国义务教育发展基本均衡县评估验收。13个县（市、区）医疗集团全部挂牌，盐湖区在国务院医改新闻发布会上进行典型经验介绍。取消公立医院药品加成，药品“两票制”全面推行。对外开放步伐加快。运城机场旅客吞吐量突破144.5万人次，增长71%。航空口岸实现临时开放，运城直飞香港、泰国旅客超过2.2万人次。中国（山西）自由贸易试验区运城片区申报工作有序推进。运城经济技术开发区被列为省级跨境电子商务示范园区。混凝土外加剂走出国门，参与“一带一路”建设。全市进出口备案企业达到698家，出口产品超过1600种。强力推动精准招商，签约项目266个，总投资1729.7亿元，到位资金468亿元。

六是聚力环境整治，生态文明建设扎实推进。深入推进大气、水、土壤污染防治，持续加大环境保护力度。中央、省环保督察整改任务按时序进度完成。河（湖）长制全面建立，汾河、涑水河河道治理及汾河沿线农村生活污水处理站试点建设有序推进。全年优良断面水质达标率、劣Ⅴ类水质比例达到考核要求。植树造林力度加大，完成12万亩造林和3.5万亩退耕还林任务。

七是聚力民生改善，人民群众的获得感、安全感不断增强。不断加大民生投入力度，全年财政资金用于民生领域的支出达到249.7亿元，占一般公共预算支出的82.4%。教育事业不断发展。中心城区新建涑水联合学校等7所中小学幼儿园；高考达线人数和达线率大幅提升；产教融合深入推进，职业教育贡献力进一步提升。就业工作卓有成效。转移农村劳动力7.92万人，城镇新增就业5.95万人，城镇登记失业率2.26%。医疗卫生服务水平有效提升。城乡居民医疗保险顺利并轨，提前完成全民参保登记计划。家庭医生签约完成年度任务。运城市第一医院建成运营，市中心医院、市中医医院规培中心、市儿童医院基建项目主体封顶。文化体育事业成绩斐然。落实习近平总书记对永乐宫壁画保护批示精神，构建了永乐宫壁画全方位监测、预警、保护体系。第十八届“群星奖”选拔赛中，我市选送的《当你老了》《蒸花馍》等舞蹈全部获奖。第十五届戏曲大赛喜获两朵“杏花奖”。40项非物质文化遗产被列为省级项目。跳水运动员李政成为我市首个世界冠军。民生实事顺利推进。省、市确定的11件民生实事完成年度目标任务，兑现了向全市人民的承诺。安全稳定形势持续好转。全市各类生产经营性事故起数和死亡人数、刑事案件和治安案件发案率均实现“双下降”，社会保持和谐稳定。

八是聚力效能提升，政府自身建设有效加强。全市政府系统按照党的建设“两个走在前”要求，深入学习贯彻习近平新时代中国特色社会主义思想和党的十九大精神，扎实开展“两学一做”学习教育和“维护核心、见诸行动”主题教育，认真落实全面从严治党主体责任，加强党风廉政建设和反腐败斗争，出台《运城市政府领导班子工作规范30条》，推动落实中央八项规定精神和国务院“约法三章”常态化、长效化。坚持依法行政，严格重大行政决策合法性审查，积极配合推进人大立法工作，政府法律顾问实现市县乡三级全覆盖。主动接受人大、政协监督，全年办理市人大代表建议241件，办复率和满意率均达100%；办理政协委员提案272件，办复271件，满意率97.8%。政府“放管服效”改革扎实推进。创新督查督办机制，建立了“13710”线上线下督查机制，有效推动了各项工作落实。同时，国防建设、军民融合取得新成效，双拥模范城建设进一步深化。统计、人防、科普、档案、地方志、民族宗教等工作和防震减灾、妇女、儿童、老龄、残疾人等事业都取得了新成绩，60家单位获国家、省271个荣誉奖项。

各位代表，过去一年，我们之所以能在转型综改任务繁重、环保压力加大的情况下取得经济逆势增长、社会和谐稳定的好成绩，这是全市上下深入学习贯彻习近平新时代中国特色社会主义思想的结果，是省委、省政府和市委坚强领导的结果，是全市上下团结拼搏、共同奋斗的结果，是市人大、市政协大力支持和社会各界有效监督的结果。在此，我代表市人民政府，向全市人民，向各民主党派、工商联和无党派人士，向各位人大代表、政协委员，向驻运部队、武警官兵和公安干警，向所有关心支持运城改革发展的各界朋友，表示崇高的敬意和衷心的感谢！

安不忘危、兴不忘忧。我们清醒地看到，我市发展仍处于爬坡过坎的关键时期，不平衡不充分问题十分突出，解决结构性体制性素质性总量性矛盾任重道远。主要表现在，转型发展任务艰巨，传统产业改造升级不快，新兴产业发展不足，大项目好项目依然不多；农业现代化水平不高，标准化、基地化、企业化、集群化发展滞后，农产品深加工层次较低，农民职业化刚刚起步；“三大收入”问题较为突出，城乡居民人均可支配收入和一般公共预算收入与全国全省平均水平还有较大差距；城乡一体化发展步伐不快，县域经济发展不平衡，基础设施建设亟待加强；环保形势依然严峻，去年能耗和大气环保指标与预期尚存差距，今后污染防治压力会进一步加大；民生和社会事业欠账较多，脱贫攻坚任务艰巨；政府职能转变还不到位，部分干部的思想解放程度不够，营商环境还不尽如人意，等等。这些问题需要我们在今后的工作中高度重视，采取有效措施，认真加以解决。

各位代表，过去一年的工作使我们深切体会到，做好政府工作：必须坚持党的领导不动摇，提高政治站位，坚定不移推动中央和省市各项决策部署落地见效；必须坚持转型发展不动摇，持续不断发力，坚定不移走出无煤少煤市转型发展新路子；必须坚持做优做强实体经济不动摇，深入推进“三个发展计划”，不断壮大市域经济实力；必须坚持改革开放不动摇，强化创新驱动，增强内生动力，拓宽开放空间，不断释放发展活力；必须坚持以人民为中心的发展思想不动摇，坚定不移把增进民生福祉作为改革发展的出发点和落脚点，不断提高人民群众的获得感和幸福感。我们坚信，只要高举习近平新时代中国特色社会主义思想伟大旗帜，以对党和人民高度负责的精神，牢固树立“功成不必在我”的政绩观，打基础、谋长远，咬定目标、久久为功，就一定能够在全省转型综改、创新驱动的竞相发展中走在前列！

微信图片_20180408154543.jpg

二、2018年工作安排

2018年是全面贯彻落实党的十九大精神的开局之年，是改革开放40周年，是决胜全面建成小康社会、实施“十三五”规划承上启下的关键一年。我们要以习近平新时代中国特色社会主义思想为指导，全面深入贯彻党的十九大精神和中央、省委、市委各项决策部署，坚持稳中求进工作总基调，坚持新发展理念，紧扣社会主要矛盾变化，按照高质量发展的要求，统筹推进“五位一体”总体布局和协调推进“四个全面”战略布局，围绕建设“示范区”“排头兵”“新高地”三大目标，以“改革抢先机，发展站前列，各项工作创一流”为总要求，以“走进新时代，建设大运城”为总抓手，紧紧扭住“转型综改、改革开放、实体经济、项目建设”四个重点，坚持深化供给侧结构性改革和深化转型综改试验区建设相结合，坚决打好“防范化解重大风险、精准脱贫、污染防治”三大攻坚战，推动质量变革、效率变革、动力变革，加强和改善民生，努力走出无煤少煤市转型发展的新路子、内陆地区对外开放的新路子和新型工业化、城镇化、农业现代化融合发展的新路子，推动全市经济增长保持中高速、全省排位保持中上游、产业发展迈上中高端，促进经济社会持续健康发展。

全市经济社会发展的主要预期指标是：地区生产总值增长7%左右，规模以上工业增加值增长8.5%，固定资产投资增长9%，社会消费品零售总额增长7%以上，外贸进出口总额增长4.5%，一般公共预算收入增长6.5%，城镇居民人均可支配收入增长7%左右，农村居民人均可支配收入增长7%以上，居民消费价格涨幅控制在3%，城镇登记失业率控制在4.2%以内。约束性指标不折不扣完成省定目标任务。围绕上述目标，重点抓好以下工作。

（一）全面强化政治引领，不忘初心牢记使命，把牢政府工作正确方向

深入践行习近平新时代中国特色社会主义思想。全市政府系统要高举习近平新时代中国特色社会主义思想伟大旗帜，把学习贯彻习近平新时代中国特色社会主义思想作为首要政治任务，坚持用这一光辉思想武装头脑。牢固树立“四个意识”，不断增强“四个自信”，坚定理想信念，提升理论素养，提高实践能力，以高度的政治自觉思想自觉行动自觉，全面贯彻党中央、国务院各项方针政策，书写新时代中国特色社会主义运城经济社会发展新篇章。

全面贯彻省委省政府各项决策部署。按照省委“一个指引、两手硬”思路和要求，围绕省委、省政府确定的建设“资源型经济转型发展示范区”、打造“能源革命排头兵”、构建“内陆地区对外开放新高地”三大目标，充分利用运城产业多元、资源丰富、区位优越等比较优势，率先在乡村振兴、工业转型和三产提质上实现新突破，率先在能源有效转化利用和新能源发展上作出新贡献，率先在发展外向型经济和对接“一带一路”国际合作上体现新成效，努力在全省转型发展、竞相发展的大格局中趟出符合运城市域特征的新路子。

坚决落实市委各项部署要求。全面落实市委四届三次、四次全会精神，统筹推进“一区五带”产业发展总布局，打好“七张牌”，推进转型综改向深处走；强化“三个发展计划”，推进实体经济向实处走；加速对外开放，推进运城发展向国际走；狠抓转型项目建设，推进产业向高端走；落实党建“两个走在前”，推进政府系统党的建设向一流走。“干”字当先、“实”字为要，干在实处，走在前列，全面推进经济社会发展实现新突破。

（二）全面深化供改综改，把握高质量发展要求，加快新型工业化进程

改造提升传统产业。坚决淘汰落后。聚焦煤炭、钢铁、电力、水泥、焦化等传统产业领域，主动淘汰不能适应市场竞争及绿色环保要求的落后产能。大力推动技术改造。实施传统产业技术改造三年专项行动，每年实施100个重点技改项目，加速技术产品更新换代，促进产业由中低端向中高端发展。全面推进智能制造。大力实施“两化”深度融合和智能制造三年提升行动，推动生产设备智能化、生产过程自动化、企业管理信息化、供应链条网络化。遴选50家骨干企业作为“两化”融合贯标试点，遴选10家龙头企业作为智能制造试点，积极争取国家支持。着力培育人工智能、增材制造、工业互联网、工业大数据产业，推动建设数字化工厂、数字化车间。着力推进绿色发展。实施环保提标专项行动，废水达到地表水Ⅴ类排放标准，废气排放全部实现在线监测，废渣“吃干榨净”，建设绿色化园区、花园式工厂，推动循环产业全链条发展。

培育壮大新兴产业。大力发展装备制造、新材料、新能源、现代化工、生物医药、电子信息、通用航空等战略性新兴产业，实现产业集聚集群集约发展。装备制造产业，走集群化、全配套路子，加速推进以汽车和轨道交通为引领的产业链建设。重点抓好大运新能源汽车园、运城经济技术开发区装配式建筑产业园、运城制版产业园、永济机电产业园建设，推动形成以汽车与新能源汽车、轨道交通、装配式建筑、印刷包装、风机泵机等为主的装备制造产业集群。新材料产业，走绿色化、低能耗路子，重点建设河津、永济、闻喜、平陆、盐湖、垣曲、芮城等地镁铝合金、铜合金、稀土永磁、纳米材料、电子信息材料、工程塑料等新材料产业园和产业链条，构建华北地区新材料产业集聚区和增长极。新能源产业，走市场化、多类型路子，大力提升新能源、清洁能源、可再生能源比重。重点推进芮城光伏领跑计划50兆瓦二期、垣曲抽水蓄能电站等一批大型新能源产业项目建设，大力发展分户式光伏发电、生物质发电和垃圾发电，加快形成水火互补、风光互济的能源格局。现代化工产业，走精细化、全循环路子，调整优化品种结构，加强河津、稷山、新绛、临猗等现代煤化工产业园建设，向精深加工延伸，促进化工产业高质量发展。生物医药产业，走品牌化、有特色路子，做强做大亚宝药业、石药银湖、华康药业、宏光医玻等骨干企业，发挥“丁桂”“亚宝”“立效”“双人”等品牌优势，强化中药产业链条配套，壮大中医药产业集群。电子信息产业，走专业化、高精尖路子，培育壮大寰烁科技、运城中兴等一批信息技术龙头企业，抓好大数据产业园建设，培育电子设备制造、电子材料、软件服务、信息安全等新一代信息技术产业。通用航空产业，走集成化、宽领域路子，加快推进芮城、绛县、万荣通用机场建设，引进战略合作者，发展航空高端铝镁合金、通航飞行器制造以及短途运输、低空旅游、环境监察、应急救援等通航服务产业链，助力区域经济社会发展。

着力打造“三大基地”。国家级铝镁合金产业基地，积极争取国家、省政策支持，推进铝镁合金创新中心建设，积极争取煤电铝材一体化改革试点，推动铝镁合金制品向工业级产品、信息家电产品、航空航天航海等军民融合领域发展。中西部精品钢基地，强化改造、扩能、提质、延伸，吸引下游企业入驻，促进产品高端化、终端化、链条化。重点抓好山西建龙140万吨冷轧、50万吨无缝钢管，高义160万吨优特带钢等项目，实现钢铁产业新跨越。汽车和新能源汽车基地，以大运汽车为龙头，加快推进新能源汽车项目实施，加速诺维兰汽车服务链条拓展，形成整车生产、零部件配套、检验检测、汽车改装维修及配套服务产业体系，构建运城汽车工业城。

做优做强实体经济。进一步创优各类政策，持续抓好“三个发展计划”，推进实体经济实现新突破。紧盯发展目标不放松，实现发展成效新突破。“龙腾虎跃”计划，加速推进170个转型项目的“千亿行动”，力争年内再实现2家企业进入龙榜，8家企业进入虎榜。“群星灿烂”计划，搭建双创平台，打造创客空间，新建10个双创基地。大力推进“小升规”，全年新增50户，净增20户。实施中小企业新技术、新产品、新项目300亿元的投资计划。建设中小企业服务平台，提升中小企业规范化运营管理水平。“凤还巢”计划，突出“抓两头、带中间”，抓大规模劳务输出、打造劳务品牌，实施“暖心、舒心、放心”行动，建立健全市县乡三级服务机构和服务机制，推动本土人才回归、吸引运城籍人才返乡创业。注重“三个转变”，实现推进方式新突破。推动“三个发展计划”从营造环境向提高实体经济发展的质量和效益转变，从党委政府外力推动向激发实体经济内生动力转变，从党委政府搭台向全社会共同推动转变。落实“四个更加”，实现环境支撑新突破。按照政策更加有效、措施更加有力、服务更加精细、方法更加创新的要求，强化组织领导，抓好统筹协调，深化包联服务，创优营商环境，全力支持实体经济发展。积极构建新型政商关系，支持引导民营经济持续健康发展。

狠抓转型项目建设。全面落实全省转型项目建设年各项工作部署，全力推动总投资3053.4亿元的1002个项目建设，落实年度投资计划826.7亿元。充分发挥开发区主战场作用，实行项目直通车制度，推进并联审批，简化办事流程，集中力量实施一批事关全局的重大牵引性项目，确保项目建设效率效果效益。我们就是要横下一条心，拧成一股劲，用项目建设的成效撑起转型发展的风帆，以实体经济的壮大挺起市域经济的脊梁！

（三）全面推进乡村振兴，落实八大提档升级行动，打造现代农业强市

全力推进“八大提档升级行动”。农业基础设施建设提档升级行动，推进农村土地整治和高标准农田建设，加强农田水利建设，推进农机装备转型升级，大力度改善农业生产条件。农业科技创新能力提档升级行动，加强农业产学研合作，加快农业科技研发和成果转化，建立健全科技服务体系，推进产业改造升级。特色农产品优势区提档升级行动，优化农业生产力布局，推动特色农产品向优势区域集中；突出特色亮点，推动规模化生产，打造一批有影响力的区域农产品品牌。优势农产品出口提档升级行动，加快优质农产品生产、检疫检验、出口贸易营销“三大体系”建设，促进更多运城农产品走向国际市场。农产品加工业提档升级行动，引进国内外知名农产品加工龙头企业，推动农产品就地转化，提高农产品附加值和市场竞争力。畜牧业规模养殖提档升级行动，以种养结合、农牧循环发展为方向，开展规模养殖示范场创建活动，加快重点项目建设，推动畜牧养殖提质增效。农业绿色发展提档升级行动，坚持以绿色生态为导向，加强农业面源污染防治和畜禽粪污无害化处理，推广生态农业，加快建立绿色低碳循环的农业产业体系。农业社会化服务提档升级行动，发展多种形式适度规模经营，大力培育家庭农场、合作社、龙头企业、农业产业化联合体等新型农业经营主体；培育各类专业化市场化服务组织，进一步提高农业生产效率。

筑牢产业兴旺基础。优化现代农业产业体系。深入实施“藏粮于地、藏粮于技”战略，保障粮食安全，粮食产量稳定在25亿公斤以上。扩大设施蔬菜、特色露地蔬菜规模，新创建5个省级标准园。全年完成核桃、杜仲等特色经济林10万亩，实施干果经济林提质增效工程23万亩。加速推进牧原500万头、温氏140万头生猪养殖项目。加快农产品深加工企业的整合、引进，大力开发功能食品，重点扶持福同惠、格瑞特、维之王、山里红、天之润等企业做大做强，年销售收入突破290亿元。加强与中国农大、山西农大、山西农科院、陕西杨凌高新示范区等院校、基地合作，支持F型杂交小麦、巨菌草、碳能肥、双季槐深加工技术等科技创新项目，主要农作物良种覆盖率达到98%以上。完善现代农业生产体系。发挥尊村、夹马口等大型引黄灌区作用，推进北赵二期和小浪底引黄水利工程，抓好末级渠系建设，全年增加灌溉面积11.5万亩。提高农业机械化水平，主要农作物耕种收综合机械化率达到73.1%以上。补改结合，每个县（市、区）年内至少完成800亩补充耕地任务。继续开展500万亩耕地质量提升工程。大力发展有机旱作农业。积极构建现代农业经营体系。大力实施省级农产品出口平台战略，创建“运城苹果”特色农产品优势区，新建20个标准化示范基地。与新华社共同设立并发布运城“苹果价格指数”，构建运城苹果网，努力提升“运城苹果”知名度和市场竞争力。推进农产品出口服务中心建设，全年农产品出口企业新增20家，达到50家，出口量达到4.5亿公斤。精心筹备第三届山西（运城）国际果品交易博览会。抓好新型经营主体培育，全年培育省级示范社35家、市级50家。加快智慧农业云平台建设，着力打造省内“互联网+农业”战略高地。完善社会化服务体系，发挥供销系统“惠农服务中心”作用，每个县（市、区）培育3—5个高水平综合性社会化服务组织。

建设生态宜居新农村。持续深化农村环境综合整治。深化“四治六化一创”专项治理，推进农村“改水、改路、改能、改房、改厕”，清理“柴堆、粪堆、煤堆、料堆”，实施衣被叠好、柴草堆好、畜禽管好、卫生搞好“四好工程”，提升农村环境品质。加强农村基础设施建设。新建改建“四好”农村公路560公里，实施安全生命防护工程500公里。解决10万农村群众饮水安全问题。加快新一轮农村电网改造升级。基本实现行政村光纤宽带网络全覆盖。高标准建设100个美丽乡村。

大力培育文明乡风。加强农村精神文明建设，深入挖掘河东文化，培育文明乡风、良好家风、淳朴民风。完善广播电视村村通、农村电影放映、农家书屋、文化活动室等重点乡村文化设施。深入开展移风易俗行动，遏制大操大办、厚葬薄养、人情攀比、高额彩礼等陈规陋习。50%的行政村建立村规民约、村民议事会、道德评议会、乡贤理事会、红白理事会、禁毒禁赌协会。加强传统村落保护，让人们记得住乡愁，留得下回忆。

持续增加农民收入。深化农业农村改革。落实第二轮土地承包到期后再延长30年的政策。深化“三权分置”改革，全面完成农村承包地确权登记颁证，促进土地有序流转。深入推进农村集体产权制度、林权制度、水权制度、供销社改革等重点改革任务，加快推进集体经营性资产改革，推动“资源变资产、资金变股金、农民变股东”。加强职业农民培训。实施职业农民培训全覆盖工程，精准培训专业技能农民10万人次。依托中农乐等资源，筹建农民职业大学，全年培育职业农民6000人。落实好强农惠农政策。落实耕地地力保护补贴等政策，加大涉农产业保险覆盖面和赔付力度，引导财政、金融、社会资本和各类人才投身乡村振兴，实现生产要素“上山下乡”。

提升乡村治理水平。加强农村“三基”建设，健全自治、法治、德治相结合的乡村治理体系。严厉查处土地征收、涉农资金、“三资”管理等领域侵犯农民权益的腐败问题。广泛开展道德模范、身边好人等评选表彰和宣传活动，弘扬真善美，传播正能量。

农业农村天地广阔、大有作为。我们将发挥运城农业资源优势，加快农业农村现代化步伐，真正让农业有干头、农民有盼头、农村有看头，让美丽乡村成为大运城的亮丽风景线！

（四）全面促进融合融通，培育壮大支柱产业，构建现代服务产业体系

培育壮大文化旅游业。挖掘“六大文化”资源和丰富的自然资源，加强顶层设计，发展全域旅游。新建改建沿黄河旅游公路200公里，统筹推进沿黄河8个县（市）旅游片区开发建设，打造黄河全景慢游走廊和自驾游目的地。继续深化旅游景区体制机制改革，筹建文化旅游发展基金和文化旅游产业发展公司，引进一批有实力、有影响的大集团投资开发旅游项目。推进盐湖、关帝庙、鹳雀楼、李家大院核心景区建设，打造5A级景区。推进关圣文化建筑群申遗工作。加强黄河金三角旅游合作，实现三省四市“一证游”。完善基础设施，推进景区“厕所革命”，强化旅游市场综合监管，打造优质旅游服务环境。大力发展康养产业，加快建设一批康养小镇、康养度假村。加快智慧旅游建设。办好美丽乡村百花节、关公国际文化旅游节等重要活动，加大旅游宣传推介力度，推进运城由旅游资源大市向旅游服务强市转变。

做大做强文化产业。继续培育国家级、省级文化产业示范基地，建设5—10个市级文化产业示范基地。重点发展包装印刷、工艺美术、文化创意、演艺娱乐等主导产业，重点培育制版集团、精睿印刷、宇达集团、澄泥砚、本命年等龙头企业，做强市文工团、蒲剧团、横店影院等演艺娱乐经营主体。加强文物保护利用和非物质文化遗产传承利用，推动文旅商融合发展。

加快发展金融、信息、物流等现代服务业。做优做强金融服务业。优化金融生态环境，加强银企对接，壮大政府性融资担保平台实力，稳步推进农信社改制，筹建各类新兴产业发展基金，支持新兴产业发展。推进大运汽车、阳光焦化、寰烁科技等企业上市进程，遴选一批上市后备企业，年内新增“新三板”挂牌企业2家以上。大力发展信息服务业，建设大数据平台和大数据产业园，发展云计算、物联网、移动互联和电子商务，促进信息消费。大力发展现代物流业，加快推进农产品国际交易服务中心、铁路物流中心、运城经济技术开发区电子商务产业园等现代物流项目，积极发展供应链物流、智慧物流、冷链物流。提高“铁公机”“岸港网”的综合承载能力，使运城成为现代物流区域枢纽。

（五）全面实施创新驱动，激发内生发展动力，持续增强发展竞争能力

推进企业创新体系建设。强化企业技术创新主体地位，实施企业科技创新提升行动，推进骨干企业国家级、省级技术中心、工程（技术）研究中心、重点实验室建设，强化铝镁合金新材料与深加工、机电与新能源汽车等装备制造、生物医药、大数据、现代农业等重点领域关键技术攻关，掌握一批核心技术，促进产业提档升级。加快众创空间和科技企业孵化器建设，推进高新技术企业“倍增计划”，建立科技型中小企业和高新技术企业培育库，全年新增高新技术企业15家。

深化产学研合作。与知名高校和国家级科研院所合作，在重点产业、医疗卫生等领域再建设2—3个院士专家工作站和2—3个博士后科研工作站。与西安交通大学合作，建立国家成果转化基地，为全市科技创新作出示范和引领。

推进科技成果转化。加强科技大市场建设，年技术合同交易额争取突破10亿元。推动设立市县两级科技成果转化引导基金，调动企业承接科技成果转化的积极性，支持企业申请专利，加强知识产权保护，强化知识产权法律援助，形成尊重知识、尊重人才、尊重智力劳动价值的浓厚氛围。

强化人才培养体系建设。落实好人才激励政策和全市百名高端人才引进计划，解决好高端人才及其亲属住房、教育、医疗等后顾之忧。支持高校调整专业设置，培养一大批适应转型发展需要的科技人才。加强职业培训，培养高技能人才。

加快智慧城市建设。着力推动网络基础设施建设，加大网络提速降费力度，出口带宽总容量达到2800G，4M宽带普及率全覆盖，60%城镇用户达到50M，80%农村用户达到20M，实现4G城乡全覆盖，推进5G应用前期工作。全面推进网络交互业务，智能终端普及率达到70%。深化智慧城市建设，全面推进“一平台五应用”，开展智慧城市二期工程建设，推进文化旅游、交通、环保、水务、安监等社会民生领域智慧应用，开展智能家居和智慧家庭服务，形成泛在、互联、融合的智慧城市体系，全面提升城市发展品位。

（六）全面深化改革攻坚，着力解决发展瓶颈，营造“六最”营商环境

深化开发区改革。强化开发区经济发展主战场和招商引资、项目建设主阵地的功能定位，对标国内先进开发区，全面完成“三制”改革，深入推进“三化”改革。加快推动运城经济技术开发区升级为国家级开发区，抓好闻喜、永济、河津等新设开发区建设，支持盐湖工业园区申报国家级高新区，加快其它开发区申报设立、扩区。按照“能放尽放、能授尽授”的原则，切实向开发区下放权力，确保授权到位。完善基础配套设施，特别是做好土地利用规划，解决好土地占补平衡，确保入驻项目一律“熟地”供应。完善各类公共服务平台，吸引人才、科技、资金等要素资源向开发区集中集聚，全面推进开发区在项目建设、产业转型上实现新突破。

强化国资国企改革。着力解决好永济纺织厂和山西农药厂等历史遗留问题，解决企业分离办社会职能和厂办大集体问题，年底前全面完成“三供一业”的移交。推动党政机关与所办企业脱钩改革。加快推进市属国有企业改革，鼓励与央企、省企、民企开展混合所有制改革，确保国有资产保值增值，增强企业市场竞争力。加快“僵尸企业”退出，妥善做好人员分流。

推进民营经济改革。实施民营经济改制工程。引导推动民营企业加快改制步伐，逐步建立现代企业制度，推动股权多元化，提升民营企业市场化竞争和可持续发展能力。实施管理提升达标工程。组织开展现代企业培训，培养造就一支优秀企业家队伍。搭建民营经济服务平台，提供改制、财务、资本、管理、人力资源等方面的专业化服务，提升民营企业管理水平。实施资产证券化工程。加强与金融证券机构合作，优化金融资本服务，着力推动企业兼并重组，解决融资信贷问题，帮助企业做强做大。实施信用等级认证工程。探索建立民营企业信用等级评价认证体系，让信用良好的企业拥有参与市场竞争的“身份证”和争取金融支持的“通行证”。实施品牌工程。大力弘扬工匠精神，全面开展质量提升行动，加强本土企业品牌培育，积极争创中国驰名商标，不断提高我市民营企业、拳头产品的品牌效应和市场竞争力。

开展招商体制改革。完善招商引资工作机制，建立专业化和市场化招商队伍。设立北京、上海、广州等重点区域招商引资办事处。创新招商引资奖励机制，开展委托招商、平台招商、以商招商、以企招商、小分队招商等多种招商模式，组织大型招商推介会，全面拓宽招商渠道。健全县级招商机构与机制，强化招商考核激励，确保招商实效。

狠抓“放管服效”改革。全面推行企业投资项目承诺制改革，全面实施市场准入负面清单制度，实行“证照分离”改革。保护市场主体权益，释放企业发展活力。大力发展“互联网+政务服务”，最大程度简化办事流程，让数据多跑路，让群众和企业少跑腿，全力打造“六最”营商环境。

精心抓好政府机构改革。全面贯彻落实党中央、国务院和省委、省政府机构改革部署要求，在市委坚强领导下，高位推动，周密部署，如期高质量完成政府系统机构改革任务。强化政治站位和使命担当，统筹抓好经济社会发展和机构改革工作，做到思想不乱、队伍不散、工作不断、劲头不减，实现机构改革与推动发展互促共进。同时，抓好财税、教育、医疗卫生等改革事项。

（七）全面扩大对外开放，提高经济外向度，打造对外开放新高地

深度融入国家开放“大战略”。紧抓国家“一带一路”重大战略机遇，积极参与“山西品牌丝路行”活动，推动更多经营主体走出国门。设立出口贸易服务窗口，扩大农产品出口渠道。充分发挥综改试验区、黄河金三角、中原城市群和关中平原城市群等“国”字号优惠政策叠加优势，加强同京津冀、环渤海、长江经济带的交流合作，推动黄河金三角三省四市在基础设施、产业协作、生态环境、公共服务等方面合作迈出实质性步伐。加强与世行、亚行、亚投行等国际金融组织的联系衔接，在基础设施、生态环境、产业转型等方面争取更多的支持与合作。

积极构建对外开放“大平台”。以打好“七张牌”为重点，做好“关公”和“古中国”两篇大文章，全面提升运城的知名度、美誉度和影响力。加强对外开放通道建设，打通高速公路省际断头路和省内重要连接线，加快蒙华铁路运城段建设，年内开工运城北站动车停车场扩建工程，做好运三高铁和阳运高速等项目前期工作。实施机场改扩建，尽早实现航空口岸正式开放。积极推进山西自贸区运城片区申报与创建工作，全面深化通关一体化改革，提升贸易便利化水平。

加快培育对外贸易“新主体”。加强玻璃器皿、铝镁合金、纺织面料、芦笋加工4个省级外贸转型示范基地建设，积极培育更多拥有进出口自营权的外贸经营主体，不断优化进出口商品结构，推动跨境贸易电子商务发展，力争有2家企业进出口额进入全省前10名，8家企业进入全省前50名。

不拒众流，方为江海。我们一定要树立开放发展理念，坚持“引进来”和“走出去”相结合，全面提升开放型经济水平，推动形成东融西进南联北拓的开放新格局，打造内陆地区对外开放新高地！

（八）全面加强新型城镇化建设，凸显市城区首位度，统筹城乡一体化发展

突出规划刚性引领。加快运城市总体规划编制，完成城北片区、城东片区控制性详细规划修编和城市设计，完成中心城区建筑垃圾、亮化、广告、色彩等十余个专项规划编制，完成河东街和解放路提升改造项目规划。启动并逐步推进“多规合一”，强化对城市空间、土地开发、产业布局、公益设施、城市生态等的空间管控能力。

提升市城区首位度。启动实施总投资106亿元的城市路网、生态绿化、公共服务设施、城市亮化提升、市政基础设施、城中村和棚户区、大运城城市规划等七大类54项城建重点项目。开工建设文化艺术中心，加快市民服务中心、万达广场等重点工程进度。推进樊村水库、安邑水库、八一水库、排水总干渠、姚暹渠的生态景观带和鸭子池湿地公园建设，构建中心城区大水系。增加公共服务供给，健全菜市场、停车场等便民服务设施，体育公园和一批小游园尽快向市民开放，加快创建全国文明城市和国家食品安全示范城市，全面推进“六城联创”。同时，要加强城市管理，提升城市品位。

加快盐临夏一体化发展。推进临猗、夏县撤县设区工作，争取早日获批。实施盐临夏一体化路网建设，优先发展公共交通，构建盐临夏三地半小时通达圈，加快一体化进程。

狠抓大县城建设。永济、河津、闻喜要瞄准大运城副中心城市的定位，其它七个大县城也要树立现代化城市发展和管理理念，围绕全市城镇化总体布局，强化产业支撑，加快产城融合，突出文化底蕴和特色设计，加强公共基础设施建设，不断完善城市功能，优化提升管理水平，把县城建设好、管理好、发展好。

打造特色小城镇。以71个建制镇为龙头，带动全市所有乡镇立足自身产业基础和比较优势，在完善功能、彰显特色上下功夫，努力培育一批现代农业、工业制造、创新创业、电子商务、旅游观光、生态宜居的特色乡镇。推进解州镇、东镇、陌南镇、历山镇创建全国特色小城镇。

（九）全面强化绿色发展，坚决打好污染防治攻坚战，推进生态文明建设

坚决打赢蓝天保卫战。深入开展大气污染防治专项行动，强化“散乱污”企业整治，着力抓好“控煤、治污、管车、降尘”措施落实。推进煤炭污染防治，中心城区开展禁煤区建设，稳妥推进“煤改气”“煤改电”。深入开展秋冬季大气污染综合治理攻坚，确保完成攻坚任务。深化工业源污染治理，严格落实重点行业错峰生产，推进提标排放。抓好机动车尾气治理，强化机动车环保监督管理。强化面源污染控制，严格控制工业企业料堆站场扬尘污染，推行“绿色施工”。加强工地和道路扬尘治理，深入开展挥发性有机化合物和油烟型污染专项整治。

毫不放松抓好水体治理。坚持饮用水、地下水、流域水、黑臭水、污废水“五水同治”。推进水污染防治重点工程建设，基本消灭劣Ⅴ类水体，打赢黑臭水体歼灭战。全面推进污水管网工程建设，污水收集率达到90%以上。完善河（湖）长制工作体系，进一步压实各级河（湖）长主体责任，全面推动境内重要河流湖泊综合治理。推动河道整治和堤坝建设，打造新绛、稷山、河津、绛县、闻喜、临猗县城段河道生态景观带。建设汾河入黄口、涑水河入黄口和伍姓湖生态湿地，各县（市、区）全面建设生态湿地公园。加快汾河、涑水河沿线农村生活污水处理站建设，提标改造城镇污水处理厂，促进企业污水提标排放，全面改善水质，进一步净化河流湖泊，让运城境内的河流水清岸绿、处处是景。

扎实推进土壤污染防治。针对农用地和重点行业企业用地，开展土壤污染状况详查。完成第二次污染源普查和生态红线划定工作。强化未污染土壤保护。按照“谁污染、谁治理”原则，开展污染治理与修复。大力推进垃圾分类处置，促进垃圾减量化、资源化、无害化处理，加快推进垃圾焚烧发电厂建设。

全力建设绿色生态屏障。持续加大植树造林力度，全年完成植树造林16.3万亩、退耕还林11.5万亩和水土流失治理37万亩，进一步提高森林覆盖率。建设中条山北麓生态景观带。继续做好南山生态修复和绿化提升，打造夏县、盐湖、永济百公里绿色长廊。实施环盐湖禁墙保护绿廊工程。加强城乡绿化，使河东大地天更蓝、山更绿、水更清、环境更优美，让绿色成为美丽运城的“底色”！

（十）全面加强社会事业，切实保障和改善民生，不断满足人民群众日益增长的美好生活需要

坚决打好精准脱贫攻坚战。坚持精准扶贫精准脱贫方略，聚焦深度贫困，压实“两不愁、三保障”标准，找准致贫原因，因村因户因人施策，注重扶智扶志扶德相结合，确保国定贫困县平陆、省定贫困县垣曲和万荣如期摘帽，181个贫困村退出，7.3万贫困人口脱贫，实现脱贫攻坚决战决胜。加大产业扶贫力度。大力推进以“五有”为目标的体制机制建设，加快推进“一村一品”特色产业扶贫，发展壮大集体经济，大力培育新型农业产业经营主体，带动有劳动能力的贫困人口脱贫。抓好易地扶贫搬迁。聚焦人、钱、地、房、树、村、稳七个问题，完成1.7万人搬迁任务，并保证贫困户搬得出、住得好、有产业、能致富。跟进完善保障举措。攻坚期内脱贫不脱政策，新产生的贫困人口和返贫人口及时纳入帮扶范围。落实好教育、保险、救助、大病救治政策，提高农村低保标准并与国家扶贫标准有效衔接，规范程序，实现应保尽保、应纳尽纳。加强扶贫资金监管。开展扶贫领域腐败和作风问题专项治理，从严查处挪用、挤占扶贫资金等违纪违法行为。

优先发展教育事业。加大财政投入力度，启动中心城区人民路小学、逸夫小学新校区建设，全市新（改、扩）建20所公办幼儿园，认定扶持150所普惠性民办幼儿园。启动全市智慧教育二期工程。消除义务教育学校66人以上大班额班级。完成薄弱校改造工作，统筹推进城乡义务教育一体化改革。强化高中教育，让运城高考继续保持全省领先地位。支持运城学院建设中西部有特色、高水平大学。提升八大职教集团运行水平。支持和规范民办教育。加强师德师风建设。发展特殊教育、继续教育和网络教育，加快实现教育大市向教育强市跨越。

着力促进就业创业。开展就业公共服务“进校园”“进企业”“进园区”活动。完善扶持高校毕业生就业创业优惠政策，支持以创业带动就业。做好退役军人安置工作。做好下岗职工再就业工作。扩大农民工就业，全面治理拖欠工资问题。全年城镇新增就业5.1万人，失业人员再就业1万人，转移农村劳动力7万人。

全力打造“健康运城”。持续推进公立医院综合改革，巩固县乡医疗卫生机构一体化改革成果，进一步完善分级诊疗体系，促进优质医疗资源向基层群众倾斜。加快发展中医药事业。做实家庭医生签约服务，县域内就诊率达到90%左右。推进卫生城镇创建，完成解州镇、泗交镇、小梁乡等7个国家卫生乡镇创建工作。启动市中心医院二期工程建设。发挥智慧食药平台作用，加强食品药品监管，保障人民群众舌尖上的安全。

不断繁荣文化体育事业。弘扬中华优秀传统文化，培育和践行社会主义核心价值观。继续开展文化科技法制卫生“四下乡”“戏曲惠民、欢乐百姓”系列活动和公益电影放映工程。繁荣戏曲演出、广播影视、新闻出版等文艺创作，推出一批精品力作。全面加强重大活动档案管理力度。高度重视修志修史。大力发展竞技体育和群众体育，抓好第二届全国青运会圣火采集筹备工作。继续办好“五老峰登山节”等体育赛事。

健全完善社会保障体系。进一步做好社保工作。继续提高城镇退休人员基本养老金、城乡居民基础养老金最低标准。完善基本医保和大病保险制度，持续扩大跨省异地就医直接结算范围。完善城乡最低生活保障制度。落实经济困难高龄失能老人和困难重度残疾人“两项补贴”，健全农村留守儿童和妇女、老年人关爱服务体系。加快发展残疾人事业。完善住房供应和保障体系，做好公租房、经济适用房和城镇棚户区改造、农村危房改造等各项工作，保障人民群众住有所居。

坚决打好防范化解重大风险攻坚战。着力化解金融风险。严厉打击非法集资、金融诈骗等违法活动。防范化解政府债务风险，积极稳妥处置存量债务。着力化解企业担保链困局，严厉打击恶意逃废金融债务行为。打造共建共治共享社会治理格局。深入贯彻总体国家安全观，强化社会治安，加快“雪亮工程”建设。深入推进“扫黑除恶”专项斗争，依法打击和惩治黄赌毒、黑拐骗等违法犯罪活动，全面加强平安运城、法治运城建设。强化乡村、社区联防、联控、联治，防范化解社会矛盾。完善基层群众自治制度，强化社区治理。加强安全生产。完善“党政同责、一岗双责、齐抓共管、失职问责”的工作格局，坚持“管行业必须管安全，管业务必须管安全，管生产经营必须管安全”的原则，严格落实企业主体责任，督促企业做到安全责任、管理、投入、培训和应急救援“五到位”。强化隐患排查，深入推进煤矿、非煤矿山、危险化学品、冶金工贸、建筑施工、交通运输、消防火灾等重点行业专项治理。加强职业病危害源头治理。高度重视应急救援、防震减灾、气象预警和防火、防汛、防疫等工作，加强非法超限超载治理，做好各项应急预案，坚决防止重大安全生产事故、重大群体性事件、重大舆情事件的发生，全力维护社会和谐稳定。

我们要持续办好为民实事。今年我们将全面承接、认真办好省政府确定的六件民生实事：一是开展“全民技能提升工程”，对广大农村转移劳动力、城镇失业人员、企业在岗职工进行职业技能培训；二是实施残疾预防重点干预和残疾儿童抢救性康复项目；三是对国定贫困县的所有农村妇女进行免费“两癌”检查服务；四是为城乡怀孕妇女提供免费产前筛查与诊断服务；五是新建90个农村老年人日间照料中心；六是免费送戏下乡2000场。在此基础上，我们还将办好五件民生实事：一是完成城镇小街小巷道路平整、路灯照明、视频监控三项工程；二是完成乡镇垃圾中转站建设任务；三是在2018年取暖季来临前，中心城区热电联产集中供热工程建成投入使用；四是在中心城区和县城区新建改建35个高标准农产品集贸市场；五是推进公共法律服务体系建设，实现市县乡村四级服务网点全覆盖。

各位代表，我们的发展是全面的，我们的事业需要河东儿女同心共建。要大力支持工会、共青团、妇联等群团组织开展工作，发展妇女、儿童、老龄、慈善和红十字会等事业；加强气象、地震、人防、科普、经济普查等工作，做好民族宗教、外事侨务、对台等工作，继续做好对口援疆工作；支持国防和军队建设改革，完善国防动员体系，加强全民国防教育，持续推进双拥工作，加强军民互动、军地互促，着力推进军民融合深度发展，凝聚起全社会“走进新时代，建设大运城”的磅礴力量！

三、加强政府自身建设

民之所望，政之所向。我们要不断提升政府治理能力和水平，努力建设人民满意的服务型政府。

强化党的领导，践行新使命。全面贯彻党的十九大精神，牢固树立“四个意识”，坚决维护习近平总书记核心地位，自觉维护以习近平同志为核心的党中央权威和集中统一领导，始终在思想上政治上行动上同以习近平同志为核心的党中央保持高度一致。深入推进“两学一做”学习教育，扎实开展“不忘初心、牢记使命”主题教育。严守政治纪律和政治规矩，坚决贯彻中央大政方针，落实省委、省政府和市委各项部署要求，不折不扣落实到行动、体现在工作、彰显于业绩。

强化依法行政，体现新要求。深入推进依宪施政、依法行政。严格执行市人大及其常委会的决议决定，依法接受市人大及其常委会工作监督和法律监督，自觉接受市政协民主监督，主动接受社会和舆论监督，认真办理人大代表建议和政协委员提案。加大审计监督力度，全面推行内审制度，有序推进审计全覆盖。加强决策咨询工作和政府智库建设，扎实开展“七五”普法，创优法治环境。严格规范公正文明执法，加强诚信政府建设，增强政府公信力和执行力。全面推进政务公开，让行政权力在阳光下运行。

强化能力提升，增强新本领。政府系统各部门特别是主要领导干部要坚持把学习当作一种政治责任和精神追求，把调查研究作为提升能力的基本功，着力解决“不会为”问题，增强“善作为”能力，不断增强“八大本领”，践行“五个过硬”要求，创造性地开展工作，推动改革发展。

强化使命担当，展示新作为。加快政府职能转变，全力推进效能提升。健全激励约束、容错纠错机制，给干事者鼓劲，为担当者撑腰，切实优化干事创业环境。坚持“五个导向、五个重在”，切实转变工作作风，不驰于空想、不骛于虚声，只争朝夕抓落实、凝心聚力谋发展。坚决惩治不作为、懒作为和乱作为行为，在建设大运城的实践中体现政府担当，展示政府作为。

强化督查问责，落实新部署。严格执行考核指标和重点工作末位分析解剖制、重点工作承诺制、重大事项报告制“三个制度”。全面深化、拓展、延伸“13710”信息化督办制度，发挥督查“利剑”作用，强化考评问责，做到件件有着落、事事有回音。

强化廉政建设，树立新形象。认真履行全面从严治党主体责任，坚持“一岗双责”，不断推进政府系统党风廉政建设和反腐败斗争。各级政府干部要立政德、明大德、守公德、严私德，严格执行中央八项规定精神和国务院“约法三章”，驰而不息整治“四风”，树立良好的政府形象。

各位代表，新时代号角催人奋进，大运城建设时不我待。让我们更加紧密地团结在以习近平同志为核心的党中央周围，在中共运城市委的坚强领导下，不忘初心、牢记使命，同心同德、锐意进取，为谱写新时代运城振兴崛起新篇章，为决胜全面建成小康社会努力奋斗!
